# Management and Outcomes of Multiple Unruptured Cerebral Aneurysms: A Descriptive Cohort Analysis

**DOI:** 10.3390/brainsci15090973

**Published:** 2025-09-10

**Authors:** Oday Atallah, Khadeja Alrefaie, Amr Badary

**Affiliations:** 1 Department of Neurosurgery, Evangelic Hospital Oldenburg, Carl von Ossietzky University Oldenburg, 26129 Oldenburg, Germany; 2 Medical School Hannover, 30625 Hannover, Germany; 3 Kuwait Institute for Medical Specialization, Kuwait 15503, Kuwait; 19200612@rcsi.com; 4 Department of Neurosurgery, Medical University Lausitz, 03048 Cottbus, Germany; amr.badary@hotmail.com

**Keywords:** cerebral aneurysms, unruptured aneurysms, multiple, treatment outcomes

## Abstract

**Background:** Unruptured cerebral aneurysms pose a significant neurosurgical challenge due to their potential for rupture, which can lead to devastating subarachnoid hemorrhage. Advances in imaging have increased incidental detection of multiple unruptured aneurysms, necessitating tailored management strategies to balance rupture risk against treatment complications. **Methods:** We retrospectively analyzed 41 patients with 101 unruptured cerebral aneurysms, assessing demographics, aneurysm size and location, treatment modalities, and clinical outcomes. Descriptive statistics and correlation analyses examined associations between aneurysm characteristics, comorbidities, and post-treatment complications. **Results:** Most aneurysms were small (<10 mm, 48.5%), primarily located at the Middle Cerebral Artery Bifurcation (27.7%). Hypertension (56.1%) and smoking (53.7%) were prevalent risk factors. Clipping was the most common intervention (81.2%), with 41.4% of patients experiencing post-operative complications and 36.6% developing neurological deficits. **Conclusions:** This study underscores the difficulties in managing multiple unruptured cerebral aneurysms owing to diverse aneurysm characteristics and patient risk factors such as hypertension, hyperlipidaemia, and smoking. Clipping was the predominant intervention, with time customized for each individual case. Despite the occurrence of problems such as vasospasm and neurological difficulties, the majority of patients maintained functional independence. The results provide significant insights into the clinical attributes, therapeutic strategies, and outcomes for this patient cohort.

## 1. Introduction

Cerebral aneurysms present a major clinical challenge because their potential for sudden rupture can lead to subarachnoid hemorrhage and severe neurological complications [1,2]. As advances in diagnostic imaging, such as time-of-flight magnetic resonance angiography, have increased the incidental detection of unruptured aneurysms during unrelated medical evaluations [3,4], the need for effective management strategies has grown. These strategies must minimize rupture risk while avoiding unnecessary interventions that carry additional complications [1,2]. Consequently, a deeper understanding of aneurysm biology, patient-specific risk factors (e.g., hypertension, smoking), and the factors driving aneurysm growth and rupture is essential to guide optimal treatment decisions.

The management of unruptured cerebral aneurysms is particularly complex due to the wide variability in their location, size, morphology, and growth potential—each contributing to a distinct risk profile [5]. Treatment decisions must carefully balance the risks of prophylactic surgical or endovascular intervention against the natural risk of rupture, while also considering patient-specific factors such as age, comorbidities, overall health status, and life expectancy. This complexity underscores the need for an individualized, multidisciplinary, and patient-centered approach.

Emerging research has identified key patterns in patient demographics, genetic and lifestyle influences on aneurysm development, and outcomes of various treatment strategies [6,7]. This retrospective cohort study seeks to delineate the clinical characteristics, therapeutic approaches, and postoperative results of individuals experiencing multiple unruptured cerebral aneurysms. This descriptive research aims to enhance future risk classification and management procedures, emphasizing the necessity for individualized, patient-centered care in this intricate clinical context.

## 2. Methods

In this retrospective analysis, we evaluated a cohort of 41 patients with multiple unruptured cerebral aneurysms, documenting demographics, aneurysm characteristics, treatment stages, and outcomes. We assessed patient demographics, risk factors, and symptoms using descriptive statistics to establish prevalence rates and associations. Aneurysm characteristics, including size and location, were analyzed by calculating the median and interquartile range of aneurysm sizes at various cerebral locations, extracting the maximum dimension for each aneurysm from clinical records. Treatment interventions were categorized and their frequency analyzed, with further examination of the durations and intervals between successive surgeries to evaluate treatment stages. Outcomes were quantitatively assessed by tracking post-operative complications, vasospasm incidence, hospital stay lengths, and neurological deficits, utilizing the Modified Rankin Scale (MRS) for follow-up assessments. Additionally, the impact of co-morbidity combinations on treatment outcomes was analyzed by identifying prevalent co-morbid conditions and their correlations with clinical outcomes using heatmaps and statistical correlation techniques. Post-operative complications were recorded as a binary variable and included all treatment modalities (clipping, coiling, flow diverter, and WEB device). Data extraction and processing involved the use of Python (version 3.11.5) and Pandas library (version 2.2.2) for handling and analyzing clinical data, ensuring robust data manipulation and analysis. Due to the limited sample size and heterogeneity in patient and aneurysm characteristics, adjusted multivariable analyses were not feasible. We summarized categorical variables as counts and percentages with 95% confidence intervals (CIs) using Wilson’s method, and continuous variables as median (IQR) and mean ± SD where informative. Given the small cohort, we performed exploratory non-parametric comparisons only when cell sizes were adequate: Fisher’s exact test for categorical associations (e.g., hypertension vs. complications) and Mann–Whitney U for continuous–binary comparisons (e.g., length of stay vs. neurological deficit). All tests were two-sided with α = 0.05 and were interpreted cautiously without multiplicity adjustments because of limited power.

## 3. Results

### 3.1. Patient Demographics, Risk Factors and Symptoms

Our study encompassed a total of 41 patients with multiple unruptured cerebral aneurysms, a total of 101 aneurysms. The cohort predominantly consisted of female patients, accounting for 82.93% (CI 68.7–91.5) of the total, with males representing 17.07% (CI 8.5–31.3). The age of patients at the time of their first operation ranged from 32 to 78 years, with a median age of 58 years. As shown in Table 1, the analysis of risk factors revealed that hypertension (56.1%, 95% CI: 41.0–70.1) and smoking (53.7%, 95% CI: 38.7–67.9) were the most prevalent. Hyperlipidemia was also common, present in (24.4%, 95% CI: 13.8–39.3) of the patients. As for the symptoms, headaches were the most commonly reported (48.8%, 95% CI: 34.3–63.5), followed by vertigo (17.1%, 95% CI: 8.5–31.3) and visual disturbances (14.6%, 95% CI: 6.9–28.4). Notably, 17.1% of the cases were asymptomatic, discovered incidentally or during diagnostics for other conditions (Table 1). Family history of aneurysms was reported in only five patients (12.2%, 95% CI: 5.3–25.5), suggesting that the vast majority were sporadic cases. Exploratory comparisons revealed no statistically significant differences in the prevalence of hypertension, smoking, or hyperlipidemia between patients with and without a family history (all *p* > 0.3).

### 3.2. Aneurysm Characteristics

We analyzed aneurysm characteristics in terms of size, numbers, and the locations associated with the most commonly reported symptom. The analysis revealed a predominance of medium aneurysms among 41 patients, with 48 classified as medium (5–10 mm), 40 as small (<5 mm), and 13 as large (>10 mm) aneurysms. The most common locations for aneurysms were the Middle Cerebral Artery Bifurcation on the left side (MCAB left) with 15 instances, followed by MCAB right with 12, and the Anterior Communicating Artery (Acom) with 11. Aneurysm distribution in patients showed that 28 patients had 2 aneurysms, 8 had 3, 4 had 4, and 1 had 5 aneurysms (Table 2).

**Figure 1:**
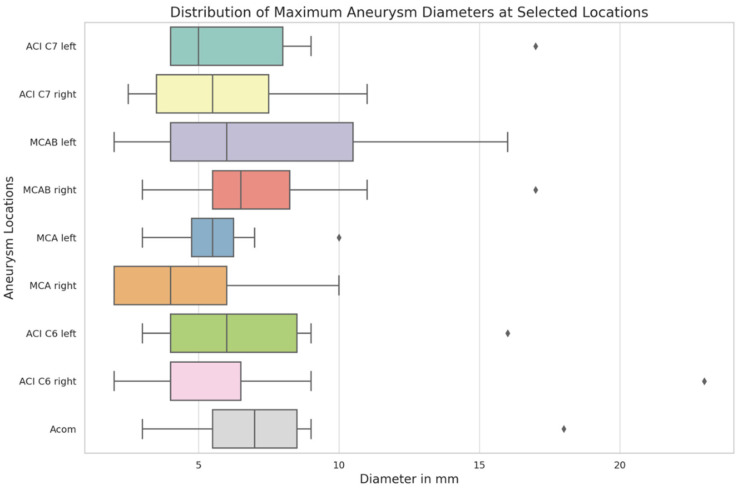
A Boxplot of The Distribution of Aneurysm Sizes at Nine Locations.

The distribution of maximum aneurysm diameters was analyzed across nine cerebral locations (Figure 1). The median diameters varied across these locations, with the smallest median diameter observed at MCA right (4.0 mm) and the largest at Acom (7.0 mm). The interquartile range showed a considerable spread in sizes, particularly in the MCAB left where the 75th percentile reached 10.5 mm, indicating a broader distribution of aneurysm sizes at this location. The Acom and both ACI C7 locations also exhibited wider variations in size, with the 75th percentile reaching 8.5 mm and 8.0 mm, respectively. Certain cerebral locations revealed notable variability. For instance, in the ACI C6 right, most aneurysm sizes were contained within a narrow range, but a significant outlier was observed, where the aneurysm diameter was substantially larger than typical values seen in this location. These outliers highlight variability in aneurysm size distributions within certain location’s, without clear implications for clinical outcomes in this cohort.

We identified multiple distinct combinations of aneurysm locations, with each combination typically unique to individual patients (Table 2). Further, our investigation into the association of headaches with these aneurysm locations unveiled a broad array of combinations, though each occurred infrequently, suggesting a diverse presentation among the study group. The most frequent combinations included locations such as ACI C7 right, MCAB left and MCA left among other combinations, which each presented in more than one patient (Table 2). Additionally, we quantitatively assessed the frequency of headaches across different aneurysm locations using a heatmap, which demonstrated that headaches are a common symptom across a wide range of aneurysm locations, with MCAB left and MCAB right being the most frequent locations, but overall no single location predominantly or significantly associated with this symptom in our cohort.

### 3.3. Treatment Stages and Interventions

**Figure 2:**
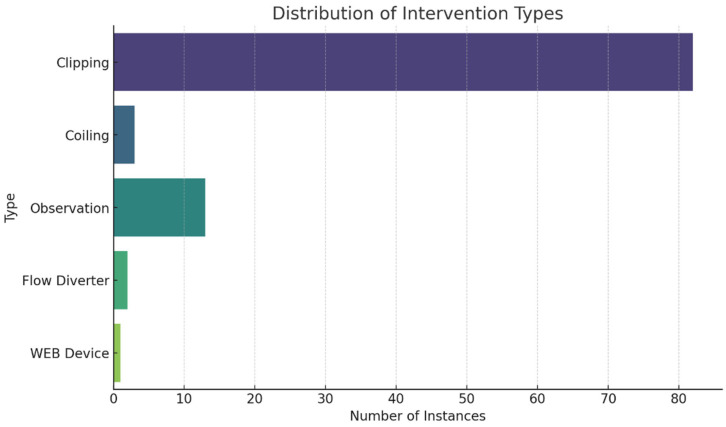
Bar Charts for Distribution of Intervention Types and Frequency of Treatment Combinations.

In examining treatment stages, 17 patients underwent a single-stage treatment, while 15 received treatment across two stages. For those needing multiple interventions, the average interval between the first and second operations was approximately 10.7 months, with a range from approximately 1.4 to 57.4 months. Only two patients required a third operation, with intervals from the second to third operation averaging about 39.2 months, showing significant variability with a standard deviation of approximately 40.4 months. In our examination of treatment interventions, clipping emerged as the most frequently applied method, accounting for 82 aneurysms. Other approaches, such as observation and coiling, were less commonly used, with 13 and 3 instances, respectively. The Flow Diverter and WEB Device were utilized in only 2 and 1 cases, respectively (Figure 2). In our analysis of treatment combinations, ‘clipping only’ approach being the most frequent, applied to 24 patients. This was followed by ‘clipping and observation’ used for 9 patients, ‘observation only’ for 4 patients, and ‘coiling only’ for 2 patients. Other combinations such as ‘clipping with WEB Device’ and ‘clipping with coiling’ were each used for one patient (Figure 2).

### 3.4. Outcome Metrics

**Figure 3:**
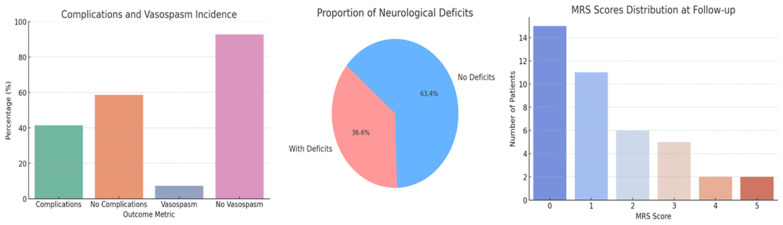
Complications and Vasospasm Incidence, Proportions of Neurological Deficits and MRS Scores Distribution.

We identified a 41.4% incidence of post-operative complications, which encompassed a range of adverse events including, but not limited to, neurological deficits, infections, and other systemic complications. We also observed a 7.3% incidence of vasospasm (Figure 3). The average length of hospital stay following the first operation was 16.8 ± 10.8 days, with a substantial range up to 70 days, and reduced to an average of 10.6 ± 4 days after the second operation (Table 3). Neurological deficits were present in 36.6% of patients post treatment, exhibiting a range of conditions including hemiparesis, aphasia, visual and gait disturbances, seizures, and cognitive impairments. The Modified Rankin Scale (MRS) scores at follow-up varied, with most patients scoring at lower levels, indicating minimal to moderate disability (Figure 3). Exploratory analyses (Table 3) were conducted to assess associations between comorbidities and outcomes. No statistically significant relationships were found, likely reflecting the limited power of the sample.

### 3.5. Impact of Co-Morbidity Combinations on Outcomes

**Figure 4:**
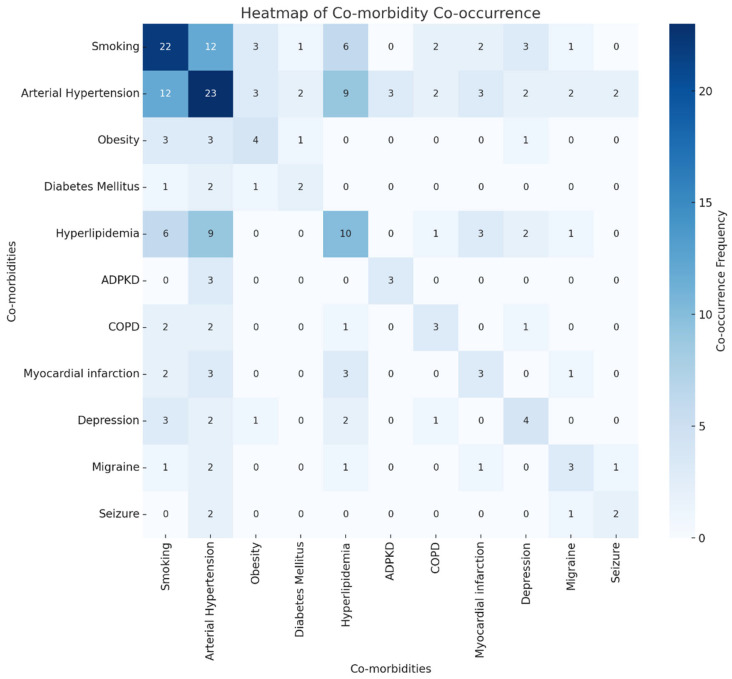
A Heatmap of Comorbidity Co-Occurrence.

**Figure 5:**
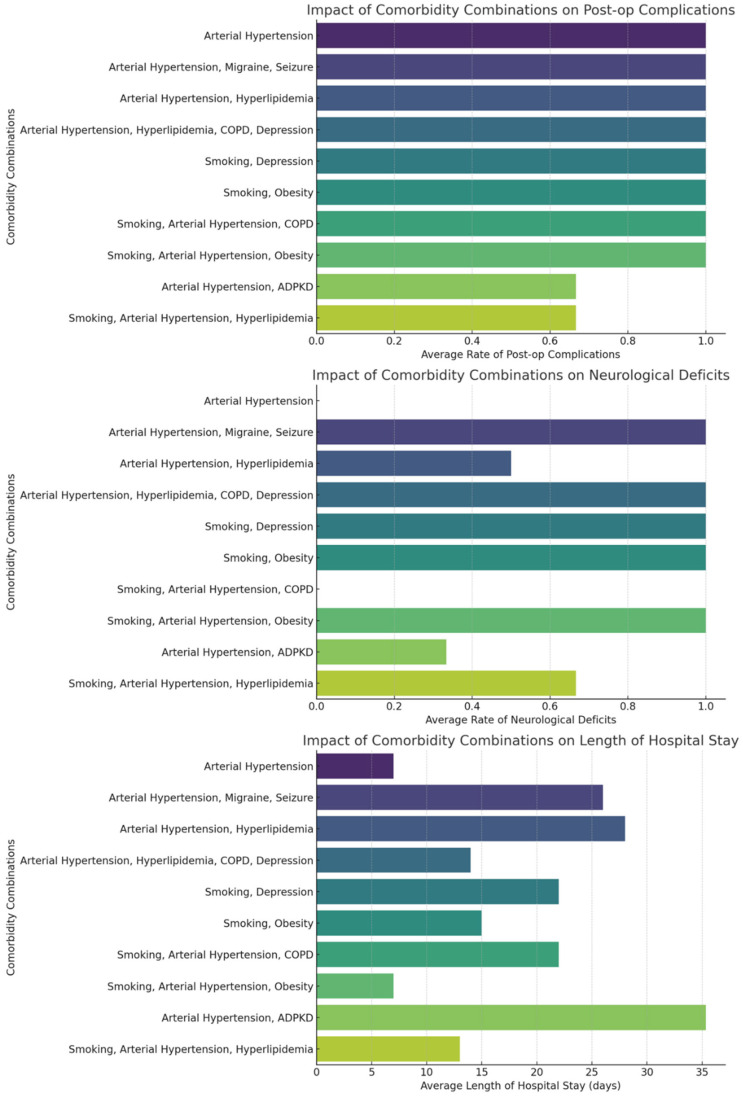
Impact of Comorbidity Combinations on Different Outcome Measures.

Our analysis of co-morbidity combinations among patients with multiple cerebral aneurysms revealed diverse impacts on treatment outcomes. The most common co-morbidities included arterial hypertension, smoking, and hyperlipidemia. The heatmap (Figure 4) visualization of co-morbidity co-occurrences indicates frequent combinations of arterial hypertension with hyperlipidemia and smoking. As shown in Figure 5, specific combinations such as arterial hypertension combined with hyperlipidemia appeared more frequently in patients with post-operative complications, neurological deficits, and longer hospital stays. We quantified the average rates of these outcomes for the top ten most frequent co-morbidity combinations, providing a detailed overview of how multiple health conditions interplay in the clinical scenario.

## 4. Discussion

### 4.1. Patient Demographics, Risk Factors and Symptoms

The predominance of female patients in our study is consistent with epidemiological data indicating a higher prevalence of cerebral aneurysms among females [8,9,10]. The high rates of hypertension and smoking further support their potential roles in the pathogenesis or progression of aneurysms [6,11,12,13]. Similarly, the frequent occurrence of hyperlipidemia as comorbidity points to a possible connection with vascular health and aneurysm stability, warranting further investigation [14]. Headache was the most common symptom, underscoring the diagnostic challenge posed by its overlap with other neurological conditions [15]. Additionally, the significant proportion of asymptomatic, incidentally discovered aneurysms emphasizes the critical role of imaging in early detection, which may guide proactive management strategies and improve outcomes. Our analysis highlights that most patients with multiple unruptured aneurysms did not have a positive family history (87.8%, 95% CI: 74.5–94.7), underscoring the predominance of sporadic rather than inherited cases in this cohort. Although family history is a recognized risk factor in the broader literature, we found no significant differences in hypertension, smoking, or hyperlipidemia prevalence between those with and without affected relatives. The low number of patients with family history (n = 5) limited the ability to detect subtle associations, and this should be interpreted cautiously. While some of these demographic and risk factor prevalence align with broader epidemiological trends, our study provides specific data from a cohort of patients with multiple unruptured cerebral aneurysms, contributing to the understanding of this specific patient subgroup.

### 4.2. Aneurysm Characteristics

The predominance of small and medium-sized aneurysms in our cohort suggests that current screening and imaging modalities are effective in detecting aneurysms at earlier, potentially less hazardous stages [16]. The frequent localization at bifurcation points—such as the middle cerebral artery bifurcation (MCAB) and anterior communicating artery (Acom)—reflects the high mechanical stress at these sites due to blood flow dynamics, which likely contributes to aneurysm formation [17]. This pattern underscores the need for targeted monitoring in high-stress vascular regions.

The variation in aneurysm sizes across different anatomical locations points to location-specific differences in aneurysm development and rupture risk [18]. A broader size range observed at sites like MCAB and the C7 segment of the internal carotid artery (ACI C7) may reflect more complex hemodynamic stressors that promote variable aneurysm growth [19,20]. In contrast, the narrower size distribution at the MCA suggests more uniform hemodynamic forces. These findings highlight the importance of incorporating location- and hemodynamics-based risk stratification into clinical assessment protocols [21]. Notably, the presence of outliers, such as the exceptionally large aneurysm at ACI C6, suggests that while most aneurysms follow predictable growth patterns, some may deviate due to unique vascular architecture, genetic predispositions, or less common environmental factors [22].

The diversity in aneurysm location combinations and their relationship with symptoms, particularly headaches, reflects the complexity of cerebral aneurysm presentations [15,23,24,25,26,27,28]. Headache in patients with unruptured intracranial aneurysms often lacks location-specific features, and the broad differential diagnosis—spanning hundreds of potential causes—mandates a comprehensive clinical evaluation, including selective neuroimaging, to guide accurate diagnosis and management [15,23,24,25,26,27,28]. The individualized anatomical distribution, without a predominant headache-associated location, suggests that headaches are a generalized symptom rather than site-specific [15,23,24,25,26,27,28].

### 4.3. Treatment Stages and Outcomes

Treatment strategies for multiple unruptured aneurysms indeed vary: many centers employ staged interventions—such as craniotomy followed by endovascular treatment—separated by a mean interval of approximately 118 days, without an associated increase in complications [29]. Similarly, second procedures have comparable hospital stays and functional outcomes to first operations. In addition, two procedures enhance curative and safe outcomes [30,31].

Clipping remains the predominant intervention, likely reflecting its well-established efficacy and suitability for the aneurysm types encountered in our cohort, as well as surgeon preference or patient-specific anatomical considerations [32,33]. Newer, less invasive techniques like Flow Diverters and WEB Devices are used less frequently, suggesting a cautious and selective approach, reserved for cases where aneurysm morphology or patient factors favor these options. The use of combined strategies—such as ‘clipping and observation’—illustrates the nuanced, individualized decision-making process in managing unruptured aneurysms, balancing the risks of intervention against conservative management based on patient and aneurysm risk profiles.

The complication (41.4%) and neurological deficit (36.6%) rates in this cohort are higher than typically reported for elective aneurysm surgery. Several factors likely contributed. First, the cohort consisted exclusively of patients with multiple aneurysms, often requiring staged or extended procedures, which inherently increase operative complexity. Second, comorbidity burden was substantial, with over half of the patients being hypertensive or active smokers, both of which are associated with poorer cerebrovascular outcomes. Third, as a tertiary referral center, our patient population may have been enriched for complex or borderline cases that are less representative of average elective practice. Finally, surgical decision-making favored clipping in the majority of cases, which, while effective, carries different perioperative risks compared with endovascular approaches.

Where documentation permitted, we distinguished transient neurological deficits from permanent deficits. Unfortunately, such details were not consistently recorded across the entire cohort, and this limitation is acknowledged. This shows the need for standardized outcome reporting that differentiates between transient and persistent morbidity, since the clinical significance of these categories is markedly different.

The observed rates of postoperative complications (41.4%) and vasospasm (7.3%) in our cohort, while seemingly high for elective procedures, underscore the inherent risks associated with surgical treatment of cerebral aneurysms, particularly in patients with multiple unruptured lesions [34,35]. In managing multiple unruptured intracranial aneurysms, the decision between single-stage and staged interventions reflects a balance between procedural efficiency and cumulative surgical risk. Single-stage approaches may increase vasospasm risk due to concentrated manipulation and endothelial stress, while staged surgeries may reduce this risk without significantly increasing length of hospital stay or functional decline [31]. Although traditionally associated with subarachnoid hemorrhage, vasospasm following elective unruptured aneurysm clipping—while rare—has been documented in 2–3% of cases, occurring days to weeks postoperatively, likely driven by intraoperative vessel manipulation and localized inflammation [36]. Additionally, the variability in hospital stay duration may partly reflect treatment modality: endovascular coiling typically leads to significantly shorter stays compared to surgical clipping, aligning with differences in recovery profiles and complication risks [37]. Neurological deficits—post treatment—are not uncommon and highlight the vulnerability of critical brain regions involved in motor, sensory, and cognitive functions [38]. Reported deficits range from hemiparesis and aphasia to cognitive dysfunction, visual disturbances, and gait abnormalities, depending on aneurysm location and surgical approach [39,40,41,42]. The occurrence of multiple concurrent deficits in some patients illustrates the potential for widespread neurological impact, emphasizing the need for multidisciplinary postoperative care, including tailored neurorehabilitation.

The distribution of Modified Rankin Scale (mRS) scores at follow-up, predominantly skewed toward lower disability levels, is encouraging, indicating that many patients maintain good functional independence. However, the presence of patients with higher mRS scores signals serious disability in a subset, necessitating ongoing medical and supportive care [43]. These findings highlight the critical need for careful risk-benefit assessment in aneurysm management, with emphasis on enhanced preoperative evaluation, intraoperative monitoring, and comprehensive postoperative care to minimize complications and optimize recovery.

While our study presents aggregate outcome data, a detailed statistical comparison of outcomes across different treatment groups (e.g., clipping vs. observation or coiling) was limited by the retrospective design and the varying characteristics of aneurysms and patients within each treatment cohort. Future research, ideally with larger, prospectively collected datasets, would enable a more robust analysis of how specific treatment modalities impact outcomes in patients with multiple unruptured aneurysms.

### 4.4. Impact of Co-Morbidity Combinations on Outcomes

Our analysis showed that the coexistence of hypertension and hyperlipidemia ap-pears to be associated with a higher frequency of postoperative complications and prolonged recovery in our cohort, although no statistical tests were conducted to establish significance. This likely reflects an underlying vascular pathology that both predisposes to aneurysm development and complicates surgical outcomes. The frequent co-occurrence of smoking with these conditions further worsens vascular health and surgical risk. Recognizing these patterns is essential for improving preoperative risk stratification and tailoring postoperative care to better address the needs of patients with complex comorbidities.

Managing unruptured cerebral aneurysms poses significant clinical challenges due to the complex balance between rupture risk and surgical morbidity. Existing tools, such as the Unruptured Intracranial Aneurysm Treatment Score (UIATS) and PHASES score, guide decisions based on aneurysm features and patient demographics but often lack integration of detailed post-surgical outcome data that critically influence treatment choices [44,45]. The American College of Surgeons National Surgical Quality Improvement Program (ACS NSQIP) offers a robust framework for surgical outcome evaluation across procedures but is not tailored to the specific nuances of neurosurgical interventions for cerebral aneurysms [46].

### 4.5. Future Perspectives

Future work should focus on refining patient selection with advanced imaging and predictive models, as well as evaluating long-term outcomes of single-stage versus staged interventions. Multicenter registries and trials are needed to establish standardized management strategies, while emerging endovascular devices should be systematically assessed in patients with multiple unruptured aneurysms.

## 5. Limitations

While comprehensive, our study has several limitations. Its retrospective design may introduce biases related to data selection and documentation. The relatively modest overall sample size (N = 41), especially within subgroups defined by specific co-morbidity combinations, naturally restricts the generalizability of our findings. Additionally, the single-center nature of the study may affect the applicability of results to other populations or clinical settings. Preoperative mRS scores were not systematically recorded across all patients, precluding direct comparison with postoperative follow-up scores. This limits the ability to assess functional decline attributable to treatment. Future studies should incorporate standardized pre- and postoperative functional assessments to enable more robust outcome comparisons. Another important limitation is the lack of detailed categorization of postoperative complications. Complications were recorded only in binary fashion, which precluded stratification into clinically meaningful subtypes such as those requiring reoperation, those managed medically, or those without long-term consequences. This limited our ability to compare the relative weight of different complication types. Future prospective registries should incorporate standardized complication taxonomies to enable more nuanced risk assessment. Future research with larger, multi-center cohorts and prospective designs is needed to validate and expand upon our observations, ensuring broader relevance and robustness.

## 6. Conclusions

The management of multiple unruptured cerebral aneurysms must evolve from fragmented decision-making toward a unified, evidence-based strategy. Our findings demonstrate that treatment success hinges on individualized planning, standardized risk stratification, and coordinated multidisciplinary care. These results can directly inform clinical decision-making by guiding the prioritization of aneurysms for intervention, tailoring surveillance imaging intervals, and optimizing follow-up protocols to detect early changes in aneurysm morphology or risk profile. To truly advance the field, future efforts must prioritize prospective multicenter collaborations and rigorous evaluation of emerging technologies. By integrating these insights into practice, clinicians can establish clear guidelines, reduce practice variability, and ultimately deliver safer, more effective, and patient-centered care for this complex population.

## Figures and Tables

**Table 1:** Patient Demographics, Risk Factors, Symptoms, Family History, and Family history vs. Key Risk Factors Exploratory Results.

Category	Metric	Value
**Patient Demographics**	Female	82.93% (95% CI: 68.7–91.5)
	Male	17.07% (95% CI: 8.5–31.3)
	Age (Mean)	56.8 ± 10.9 years
	Age (Median)	58 years
**Risk Factors/Comorbidities**	Yes	92.7%
	No	7.3%
	Hypertension	56.1% (95% CI: 41.0–70.1)
	Smoking	53.7% (95% CI: 38.7–67.9)
	Hyperlipidemia	24.4% (95% CI: 13.8–39.3)
	Depression	9.8% (95% CI: 3.9–22.5)
	Obesity	9.8% (95% CI: 3.9–22.5)
**Symptoms**	Total Symptomatic	82.9%
	Headache	48.8% (95% CI: 34.3–63.5)
	Vertigo	17.1% (95% CI: 8.5–31.3)
	Visual Disturbances	14.6% (95% CI: 6.9–28.4)
	Asymptomatic Rate	17.1% (95% CI: 17.1–31.3)
**Family History**	Yes	12.2% (95% CI: 12.2–25.5)
	No	87.8% (95% CI: 74.5–94.7)
**Risk Factor**	**Prevalence by Family History**	**Fisher *p*-value**
Hypertension	40.0% with FH (n = 5), 58.3% without FH (n = 36)	*p* = 0.64
Smoking	60.0% with FH (n = 5), 52.8% without FH (n = 36)	*p* = 1.00
Hyperlipidemia	0.0% with FH (n = 5), 27.8% without FH (n = 36)	*p* = 0.31

**Table 2:** Aneurysm Characteristics.

Characteristic	Detail	Count
**Aneurysm Size**	Small (<5 mm)	40
	Medium (5–10 mm)	48
	Large (>10 mm)	13
** Top Locations **	MCAB Left	15
	MCAB Right	12
	Acom	11
	MCA Left	9
	ACI C6 Left	8
	ACI C6 Right	7
	ACI C7 Left	7
	ACI C7 Right	6
	MCA Right	5
	Basilar	4
**Location Combination**	ACI C7 right, MCAB right, MCA left	2
	MCAB right, MCA right, ACI C6 right	2
	MCAB left, MCA left, ACI C6 left, Acom	2
	MCAB left, ACI C6 left, Pericallosal left	2
	MCAB left, ACI C6 right	2
	MCAB right, MCA left, MCA right, ACI C6 left, Acom	2
**Number of Aneurysms**	2 Aneurysms	28
	3 Aneurysms	8
	4 Aneurysms	4
	5 Aneurysms	1

**Table 3:** Outcome Parameters and Exploratory Analyses of Comorbidities and Outcomes.

Outcome Metrics		Details—Confidence Intervals (Wilson 95%)		
Post-operative Complications *		41.4% (95% CI: 27.6–56.6) with complications		
**Vasospasm Incidence**		7.3% (95% CI: 2.5–19.6) with vasospasm		
**Length of 1st Hospital Stay**		Mean: 16.8 ± 10.8 days, Range: 7–70 days		
**Length of 2nd Hospital Stay**		Mean: 10.6 ± 4.0 days, Range: 5–20 days		
**Neurological Deficits**		36.6% (95% CI: 23.5–52.0) with deficits		
**MRS Scores at Follow-up**		Distribution: 0 (15), 1 (11), 2 (6), 3 (5), 4 (2), 5 (2)		
**Exploratory analyses of comorbidities and outcomes**				
**Comparison**	**Group A**	**Group B**	**Test**	***p*-value**
Hypertension vs. Post-op Complications	With HTN: 12/23 (52%)	No HTN: 5/18 (28%)	Fisher’s Exact	0.20
Hospital stay vs. Neurological Deficits	No deficit: median 14 days (n = 26)	With deficit: median 15 days (n = 15)	Mann–Whitney U	0.29

* Complications include events after all intervention types.

## Data Availability

The data presented in the following study are available from the corresponding authors upon request. The data are not publicly available due to privacy restriction.

## Abbreviations

MCAB: Middle Cerebral Artery Bifurcation; MCA: Middle Cerebral Artery; Acom: Anterior Communicating Artery; ACI: Arteria Carotis Interna (Internal Carotid Artery); mRS: Modified Rankin Scale; UIATS: Unruptured Intracranial Aneurysm Treatment Score; PHASES: Population, Hypertension, Age, Size of aneurysm, Earlier SAH from another aneurysm, Site of aneurysm (risk score); ACS NSQIP: American College of Surgeons National Surgical Quality Improvement Program; IRB: Institutional Review Board; TOF-MRA: Time-of-Flight Magnetic Resonance Angiography; AJNR: American Journal of Neuroradiology.
